# 8-Chloro-cAMP-Related Changes on Mice Uteri

**DOI:** 10.1100/tsw.2002.282

**Published:** 2002-05-22

**Authors:** Andrea Actis, Maximo Croci, Emanuel Levin, Rosa Bergoc

**Affiliations:** Department of Human Biochemistry, School of Medicine, University of Buenos Aires, Buenos Aires, Argentina; Institute of Health Sciences, Foundation Barceló, Buenos Aires, Argentina; Institute of Immunooncology Dr. J.V. Crescenti, Buenos Aires, Argentina; Radioisotopes Laboratory, School of Pharmacy and Biochemistry, University of Buenos Aires, Junin St. 956 (1113), Buenos Aires, Argentina

**Keywords:** 8-Chloro-cAMP, tamoxifen, medroxyprogesterone, mice, uterus, histopathology

## Abstract

Histopathological effects of cAMP analog (8-Chloro-cAMP), tamoxifen, and medroxyprogesterone, alone or combined, upon BALB/c mice uteri are reported. 8-Chloro-cAMP diminished uterine weight, but did not modify its histopathology or estral cycle significantly. Tamoxifen diminished uterine weight showing cystic hyperplasia and an estral cycle arrested at diestrus. Medroxyprogesterone increased uterine weight, caused a swelling of the endometrium and a pseudopregnancy estrus. When combined with 8-Chloro-cAMP, tamoxifen or medroxyprogesterone always had a predominant effect. We concluded that the effects of 8-Chloro-cAMP on mice uteri did not cause significant changes on its histopathology, but diminished its weight.

### INTRODUCTION

The study of 8-Chloro-cAMP pharmacological effects, alone or in combination with other compounds such as tamoxifen and medroxyprogesterone on a hormone-dependent tissue like the uterus, has two objectives: first, to explore how those pharmacological agents affect the cell development of a target tissue of estrogens and, second, to reveal the diverse protein-protein interactions by the different transductional pathways activated by each of them. In this work, we studied the effects of 8-Chloro-cAMP, alone or in combination with tamoxifen or medroxyprogesterone, on the histopathology of uterine mice. 8-Chloro-cAMP is a cAMP analog drug that binds to the regulatory subunit of cAMP-dependent protein kinase (PKA), as determined by Cho Chung[1]. PKA has two regulatory (R) and two catalytic subunits. There are two kinds of R subunits: $R_I$ and $R_{II}$. $R_I$ is related to the proliferative actions of cAMP, and $R_{II}$ is related to the differentiation processes triggered by cAMP. 8-Chloro-cAMP binds preferentially to the $R_{II}$ subunit, and the antitumoral action is attributed to this binding[2]. It is known that cAMP induces an inhibitory effect on cell growth[3]. The antitumoral action of 8-Chloro-cAMP was investigated in several organs, such as the colon, in breast cell lines cultured *in vitro*[4], in nude mice[5,6,7], and using *in vivo* models[4]. These investigations determined that 8-Chloro-cAMP inhibits autocrine and angiogenic growth factor production in colorectal and breast cancer cells. Scala et al.[8] and other researchers[9] investigated the mechanism of the action of this drug. Currently, 8-Chloro-cAMP is being used as an anticancer drug in various treatment protocols[1,6,10]. Medroxyprogesterone is a synthetic progesterone-analog and, as such, binds to progesterone receptor (PR) molecules. As indicated by Panutti et al.[11], it is used in mammary cancers to counteract estrogen action. In mice, Lanari et al.[12] and Molinolo et al.[13] showed that medroxyprogesterone was able to induce mammary carcinogenesis and accelerates tumor growth in the medroxyprogesterone-dependent tumors. Tamoxifen is a selective estrogen receptor modulator (SERM) widely utilized for breast cancer treatments as an antiestrogen, acting by different mechanisms but mainly through blocking estrogen receptor (ER) molecules[14,15]. In a previous report, we studied the effect of tamoxifen, medroxyprogesterone, and 8-Chloro-cAMP in ERs and PRs of BALB/c mice uteri[16,17]. Furthermore, we determined that 8-Chloro-cAMP and its association with medroxyprogesterone had a different effect on two mammary tumor sublines induced in BALB/c mice by medroxyprogesterone injection[18]. It was our aim to analyze the effect of 8-Chloro-cAMP, alone or in combination, with other antineoplastic agents on the histopathology of uterine tissue.

### EXPERIMENTAL MATERIALS AND PROCEDURES

#### Animals

Female BALB/c mice 50–60 days old were employed. The animals were kept under the conditions recommended by the Guide of Care and Use of Laboratory Animals, U.S.

#### *In Vivo* Treatments

Animals were randomly divided into six groups of five mice per group. One group was used as the control group. These were treated with vehicle. Mice of the other five groups received different treatments initiated simultaneously. The 8-Chloro-cAMP group received a subcutaneous pellet (15 mg/kg day); each pellet lasted 10 days and was changed successively. The medroxyprogesterone group received a subcutaneous drug depot (0.25 mg/kg day). The tamoxifen group was treated with a subcutaneous pellet (0.25 mg/kg day); drug delivering from the pellets of tamoxifen and medroxyprogesterone lasted 2 months with a linear-type releasing kinetic. In combined treatments, doses of the combination of drugs utilized were the same as previously described. The selection of dose employed was based in previous results obtained from murine mammary tumors[17].

#### Estrus Cycle and Uterine Weight Determination

The estrus cycle was determined daily by vaginal smears.

### Histopathology

Representative samples of all uteri were harvested for histological examination.

### Reagents

Gador S.A., Buenos Aires, Argentina, kindly provided tamoxifen and medroxyprogesterone. 8-chloro-cAMP was a gift from Y.S. Cho Chung, M.D., National Cancer Institute, U.S.

## RESULTS

### Uterine Weight

The drugs tested in this study acted diversely on this parameter as is shown in Table 1. This table sums up quantitative data and each treatment's significance level.

### Estrus Cycle

Table 2 shows the effects of drugs employed upon the estrus cycle.

### Histopathology

Table 3 summarizes histopathological findings according to the different treatments. See Fig. 1.

**TABLE 1**
**Changes in Uterine Weight According to Treatments**

| Treatment | Uterine Weight (mg) (Mean ± SD) | *p* value |
|---|---|---|
| Vehicle | 111.6 ± 11.1 | —- |
| 8-Chloro-cAMP | 84.2 ± 14.0 * | <0.05 |
| Tamoxifen | 62.6 ± 10.1 *** | <0.001 |
| Medroxyprogesterone | 150.8 ± 19.4 *** | <0.001 |
| 8-Chloro + tamoxifen | 62.6 ± 3.4 *** | <0.001 |
| 8-Chloro + medroxyprogesterone | 137.6 ± 6.3 * | <0.05 |

**TABLE 2**
**Changes in Estrus Cycle According to Treatments**

| Treatment | Estrus Phase |
|---|---|
| Vehicle | Normal cycle (4 days) |
| 8-Chloro-cAMP | Extended cycle duration (10 days) |
| Tamoxifen | Permanent diestrus |
| Medroxyprogesterone | Permanent pseudopregnancy aspect |
| 8-Chloro + tamoxifen | Permanent diestrus |
| 8-Chloro + medroxyprogesterone | Permanent pseudopregnancy |

**TABLE 3**
**Histopathological Findings According to Treatments**

| Treatment | Histopathological Findings | Figure |
|---|---|---|
| Vehicle | Normal proliferative epithelium | 1a |
| | Scarce stroma | |
| | Lack of secretory glands | |
| 8-Chloro-cAMP | Same as vehicle | 1b |
| Tamoxifen | Cystic hyperplasia | 1c |
| | Hyperproliferative areas | |
| | Important mitotic activity | |
| | Scarce stroma | |
| Medroxyprogesterone | Progestational-like endometrium | 1d |
| | Numerous glands with serrated appearance and secretory signs | |
| 8-Chloro + tamoxifen | Same as tamoxifen | 1e |
| 8-Chloro + medroxyprogesterone | Same as medroxyprogesterone | 1f |

### DISCUSSION

8-Chloro-cAMP is a cyclic-AMP analog directly involved in cell proliferation and neoplastic transformation that causes growth inhibition in a variety of human cancer cell types. It is also known that *in vitro* it inhibits the expression of autocrine and paracrine growth factors[19]. Recently, numerous researchers have investigated the possible dual anticancer mechanism of 8-Chloro-cAMP through inhibition of cell proliferation and induction of apoptosis[20,21,22,23]. In a previous study, we analyzed the action of 8-Chloro-cAMP, tamoxifen, and medroxyprogesterone, alone and in combination, upon the uteri of BALB/c mice[17]. We observed that 8-Chloro-cAMP and medroxyprogesterone did not modify significantly the content of estrogen receptors. In the current study, we found that 8-Chloro-cAMP did not overcome the effects seen in the treatment of mice with tamoxifen (weight loss, estral cycle arrested at diestrus phase, cystic hyperplasia, and mitogenic action) in the uterine tissue. In combination with medroxyprogesterone, the progestin aspect of the endometrium produced by medroxyprogesterone was not counteracted by 8-Chloro-cAMP, and the uterine weight was not increased either. The interrelations or cross talk between different transductional pathways, which regulate the expression of a gene, are affected by the use of drugs addressed to some of the components of those pathways. So, as a result of the cross talk, unexpected effects can be seen. 8-Chloro-cAMP as well as tamoxifen and medroxyprogesterone act on diverse transductional pathways. The combined administration of these drugs allows studying the way by which the interactions between proteins (cross talk) are modified when drugs that act only through a way are given[17]. In this article, even if 8-Chloro-cAMP effects on the analyzed parameters are not quite noticeable at the level of dose employed, we cannot reject the possibility that these effects may appear at different doses. In the mouse uterus, we determined that 8-Chloro-cAMP caused modifications in subcellular content and distribution of estrogen receptors[17]. It is interesting to comment on the modifications caused by 8-Chloro-cAMP alone on the histology of uterus, like a lower number of proliferative glands and the prolonged estral cycle; moreover, this compound diminished significantly the uterine weight ($p < 0.05$). These modifications could be signaling that by binding itself to the subunit $R_{II}$ of PKA, 8-Chloro-cAMP is able to favor differentiation and to diminish proliferation.

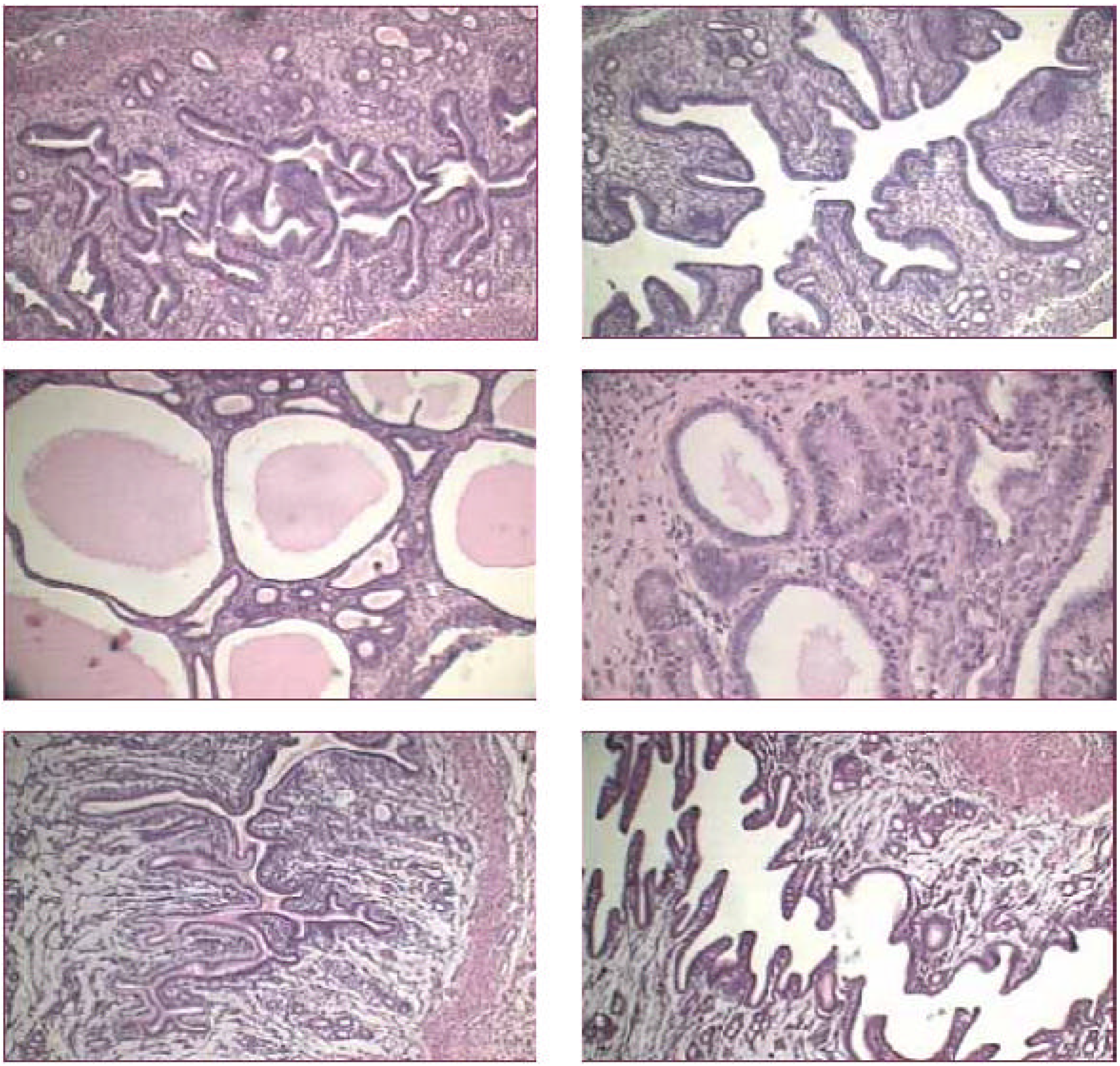

**FIGURE 1.** Endometrium slice of mouse uterus under different treatments. **1a:** Vehicle; proliferative endometrial glands with normal number of mitoses and absence of secretory changes. Dense stroma. **1b:** 8-Chloro-cAMP; proliferative endometrial glands with scarce number and scarce stroma. **1c:** Tamoxifen; quistic endometrial hyperplasia with secretory apical mucosal sectors and multistratification; mitotic figures were seen. **1d:** 8-Chloro-cAMP + tamoxifen; proliferative endometrial mucosae with multistratificated nucleous, mitoses and cystic glands. **1e:** Medroxyprogesterone; highly indented endometrial glands with marked serrated appearance; secretory vacuole located in the basal portion of the cylindrical epithelium. Clear edematous and scarcely cellular stroma between glands. **1f:** 8-Chloro-cAMP + medroxyprogesterone; important number of secretory glands with serrated appearance, scarce apical secretion, and edematous stroma. In all cases, HE 100X.

### CONCLUSION

The beneficial effects of estrogens in females are well known. However, these sexual hormones can trigger the proliferation of cells that will grow as a tumor, especially for breast cells. The first-line treatment for breast cancer that expresses estrogen receptors is tamoxifen. But this SERM, which exhibits estrogen receptor antagonism in breast cells, also presents agonist activity in the uterus. On the other hand, 8-Chloro-cAMP is able to reduce proliferation and favors differentiation acting through $R_{II}$ subunit of PKA. The evidence of cross talk between both biochemical pathways is well documented. Whether the combined treatment of tamoxifen and 8-Chloro-cAMP can keep proliferative activity triggered by tamoxifen under control remains to be determined.

### ACKNOWLEDGMENTS

This work was supported by grants from Buenos Aires University (TB069) and from the National Agency of Scientific and Technological Promotion (PICT 04656). The authors acknowledge the technical assistance of Inés Nievas (CONICET).

### BIOSKETCH

**Rosa Maria Bergoc** is a Full Professor of Radiochemistry at the Radioisotopes Laboratory and head of Physics and Mathematics Department, School of Pharmacy and Biochemistry, Buenos Aires University. She is a Member of the National Council of Scientific and Technological Research (CONICET). Dr. Bergoc holds a Ph.D. in Biochemistry from Buenos Aires University, Argentina, with Post-doc in Radiation Protection, Triest, Italy. Her research interests include the action of insulin growth factor type-1 and histamine on growth regulation of malignant cells, and its relation with cellular radiosensibily. She has received more than twenty awards for researches on biochemistry fields and educational activities. Dr. Bergoc is a Member of the European Histamine Research Society, International Radioprotection Society, Women in Nuclear Association, New York Academy of Science, and Cancer Detection and Prevention Society, among others.
